# Report of the National Vaccine Establishment

**Published:** 1812-09

**Authors:** Fr. Milman, James Hervey

**Affiliations:** President; Register


### For the Medical and Physical Journal.

REPORT *of the* NATIONAL VACCINE ESTABLISHMENT.

*To the Right Hon. Richard Ryder, Principal Secretary of State, Home Department, &c. &c. &c.*

*National Vaccine Establishment,*
SIR, *Leicester-square, March* 9, 1812.

THE Board of the National Vaccine Establishment have the honor of reporting to you, that, during the year 1811, the surgeons appointed by their authority to the nine stations in London, have vaccinated 3,148 persons, and have distributed 23,794 charges of vaccine lymph to the public. The number vaccinated this year rather exceeds that of the year 1810, and the demand for lymph has been often so great that it could not be immediately supplied.

They have great satisfaction in stating, that since the commencement of this establishment, not a single instance of the accession of small-pox, after vaccination, has occurred to any of the vaccinating surgeons of the nine stations.

The Board report, that they have been lately furnished with many satisfactory official documents from the naval and military departments of government, respecting the progress of vaccination, and have likewise obtained some other authentic papers on the subject, containing much important information. They think it expedient to lay before you a summary of their contents.

It appears, that, in consequence of an order from the Lords Commissioners of the Admiralty, vaccination has been practised in the navy to a great extent; and, although it has not been universally adopted, the mortality from the small-pox, among seamen, is already greatly diminished.

In the army, the practice of vaccination has been long established, by an order from the Commander in Chief, and its effects have been decidedly beneficial; for almost the only persons among the troops who have lately been affected with small-pox, have been either recruits, who had received the infection previous to their enlistment, or soldiers who had not been vaccinated, on the supposition of their having had the variolous disease. Thus, with a few exceptions, a disorder formerly so fatal to the troops, is now considered as nearly extinguished in the army.

By information transmitted to the Board from their numerous correspondents in all parts of the country, it appears that vaccination is almost every where gaining ground, throughout the British dominions, though its progress is very different in different places; and it is found that the number of deaths from the small-pox is uniformly decreasing, in proportion as vaccination vaccination becomes more general, and the inoculation of the small-pox declines.

The disappearance of the small-pox from the island of Ceylon, was noticed in the Report of last year; and the Board has now the pleasure of stating, from sources of authentic and satisfactory information, that, in consequence of vaccination, this dreadful disease has in no instance lately occurred in the island of Anglesey, in the populous city of Newcastle-upon-Tyne, in the town of Petworth, or in the adjoining district.

Through the different counties of England, the practice of vaccination is becoming general, and the small-pox is gradually declining; and even in London, where the opposition to the new inoculation has been most violent, it is prevailing, and its salutary effects are becoming daily more evident. At present, by the best estimate we are able to make, it appears that nearly two-thirds of the children annually born in the metropolis, are vaccinated either by charitable institutions or private practitioners; and that the number of deaths by small-pox has proportionally decreased. Previous to the discovery of vaccination, the average number of deaths by small-pox within the bills of mortality, was 2,000 annually; whereas, in the last year, only 751 persons have died of that disease, although the increase of population within the last ten years has been 133,139. The increase of population throughout Great Britain, in the same period of time, has been 1,609,000; and to these augmentations the practice of vaccination has probably much contributed.

The Reports from the Cow-pock Institution in Dublin are of the most favorable nature, and furnish sufficient reason to believe, that since the introduction of the vaccine preventive, the mortality from small-pox has considerably declined in that city. The correspondence of the Institution affords satisfactory evidence of the progressive increase of vaccination throughout Ireland. In most of the principal towns of that kingdom, the poor have the advantage of gratuitous inoculation with cow-pox, either at the hospitals, or at the houses of the physicians; and it is stated, that, among the higher ranks, vaccination is universally adopted.

The accounts from Scotland, particularly those from the faculty at Glasgow, which have been transmitted to the Board, furnish evidence of the general and rapid increase of vaccination in the northern part of the island, and give the most satisfactory proofs of the success and efficacy of the practice.

Notwithstanding the incontrovertible evidence of the very great advantages of vaccination, it is much to be lamented that that there are still some medical practitioners, though the number of them is comparatively small, who obstinately persist in disseminating by inoculation the contagion of the small-pox, and who strenuously encourage and support, especially among the lower orders of the people, the prejudices against the new practice; rumors are industriously spread abroad, of deforming and loathsome diseases produced by this practice; and numerous misstatements of cases are published, of the occurrence of small-pox after vaccination. That, in some instances, the small-pox has affected persons who have been most carefully vaccinated, is sufficiently established; nor ought we to be surprised at this, when we consider that the inoculation for the small-pox sometimes fails, and that several cases may be produced, in which persons have been affected with the natural disease more than once in the course of life. The number of instances of small-pox after vaccination, however, is very small; and we may fairly presume, that in proportion as improvements are made in the practice, such occurrences will be still more rare.

The Board have infinite satisfaction in stating the two following important and decisive facts in proof of the efficacy and safety of vaccination, viz. that in the cases which have come to their knowledge, the small-pox, after vaccination, with a very few exceptions, has been a mild disease; and that, out of the many hundred thousand persons vaccinated, not a single well-authenticated instance has been communicated to them, of the occurrence of a fatal small-pox after vaccination.

They cannot conclude their Report, without adverting to the mischiefs which are daily arising from the diffusion of the fatal contagion of small-pox in the community, in consequence of variolous inoculation, among the lower classes of the people, which constantly keeps up the contagion, and, where it saves a single life, exposes numbers to a most dangerous disease. It is greatly to be wished that this evil could be checked, by such measures as government in its wisdom might judge proper to frame, in order to prevent the spreading of the small-pox, and thus keeping up a continual source of infection in the heart of the metropolis.

The constant renewal of the contagion of small-pox in this capital, which they so deeply lament, is strikingly contrasted with the advantages enjoyed by several of the other capitals of Europe, in consequence of the universal adoption of vaccination by medical practitioners, seconded by the authority of government. The cities of Vienna and Milan, in which the mortality from small-pox was formerly more considerable in proportion to their population than in London, have been for for some time freed altogether from this destructive pest; the first for five, and the latter for eight years, according to the statement of doctors De Carrio and Sacco: and in the city of Geneva, the small-pox has been nearly extirpated. In Switzerland in general, but more particularly in Geneva, the extension of the blessings connected with vaccination, has in a great degree depended on the warm and active co-operation of the clergy, who were assiduous in recommending the practice to their parishioners from the pulpit, as well as promoting it by every other exertion in their power. Impressed with the strongest conviction of the great advantage which vaccination would derive from a similar co-operation in this country, the Board formerly considered it as a part of their duty to address the bishops, for the purpose of soliciting their assistance in checking the ravages of the small-pox, by rendering the benefits of the vaccine inoculation more extensively known.

The Board has great pleasure in stating, that the money granted by parliament during the last session has been sufficient to defray the expenses of the year 1811; and they are of opinion, that the same sum will be adequate to the expenditure of the current year.

FR. MILMAN, President.

By order of the Board. JAMES HERVEY, Register.

